# Error orientation in a decision-making simulation program: differences between promotion vs. prevention focus

**DOI:** 10.3389/fpsyg.2023.1057634

**Published:** 2023-08-24

**Authors:** Alicia Arenas, Elena Briones, Carmen Tabernero

**Affiliations:** ^1^Department of Social Psychology, Universidad de Sevilla, Sevilla, Spain; ^2^Instituto Maimónides de Investigación Biomédica de Córdoba (IMIBIC), Córdoba, Spain; ^3^Department of Education, Universidad de Cantabria, Santander, Spain; ^4^Department of Social Psychology and Anthropology, Universidad de Salamanca, Salamanca, Spain

**Keywords:** error orientation, decision-making, simulation program, promotion focus, prevention focus

## Abstract

Changing situations develop work environments where workers must generate strategies to learn and persist from continuous errors and setbacks. Previous research has shown that errors enhance motivation, break the routine, lead to creative solutions, and reduce frustration; however, this positive aspect seems to have a stronger presence if personal factors and contextual background support such a focus. The main aim of this paper was to analyse, with an experimental design, how different frames about errors and negative feedback (error promotion versus error prevention) affected performance and decision-making processes in a complex simulation task, taking into account individual attitude towards errors. The sample included 40 employees of a Spanish transportation company (37.5% were women and 62.5% were men). Firstly, participants answered a questionnaire about their individual Error Orientation. Then, they were randomly assigned to an experimental condition to carry out a complex decision-making task through a multimedia simulator, which aimed to expose the participant to factors that influence the dynamics of innovation and change, elements that are present in all modern organizations. None of the participants had previous experience in the task. Performance was measured through different aspects: (1) final performance values: adopters, points, time to make decisions and time after receiving negative feedback; (2) the decision-making process. Results showed that error orientation is related to final performance, especially error risk taking and error communication. The effect of the experimental condition was higher for the time to make decisions after receiving negative feedback and for the time to complete the simulation program. Those who worked under the error prevention condition took significantly longer to perform the task. Although our results show non-consistent effects, which frame than the other (promotion versus prevention) is better to make decisions is discussed. A promotion frame prioritizes flexibility, openness, and rapid progress, but does so by sacrificing certainty, and careful analysis. The most crucial factor may be which one best fits the demands of the task at hand.

## Introduction

The modern employment market must adapt quickly to the continuous changes that occur around us. This critical adaptation includes achieving the highest levels of motivation and personal development for all employees. To do this, it is essential to know which psychosocial factors affect management decisions, regardless of whether these decisions are erroneous or successful. During a workday, employees face new, complex, and sometimes conflicting situations to which they must respond immediately even if they do not yet have a full understanding of the situation. Changing situations develop work environments where workers must generate strategies to learn and persist from continuous errors and setbacks. Over the last three decades, the literature on this subject has shown the need to understand and accept the errors that can occur, taking them into consideration and learning to live alongside them (Keith and Frese, 2005). Furthermore, errors play a positive role in employee training because they instigate learning and the exploration of new challenges (Dormann and Frese, 1994). From this perspective, errors enhance motivation, break the routine of daily activities, lead to creative solutions, and reduce frustration; however, this positive aspect seems to have a stronger presence if personal and contextual background support such a focus. Following Lie et al. (2016) level of analysis perspective in their error literature review, not all individuals would benefit equally from a positive context toward errors. They suggest that individual traits play a moderating role in the relationship between context and performance outcomes. Thus, the main aim of this paper is to analyse, with an experimental design, how different frames about errors and negative feedback affect performance and decision-making processes in a complex simulation task, taking into account an individual factor such as the attitude toward errors.

**Figure 1:**
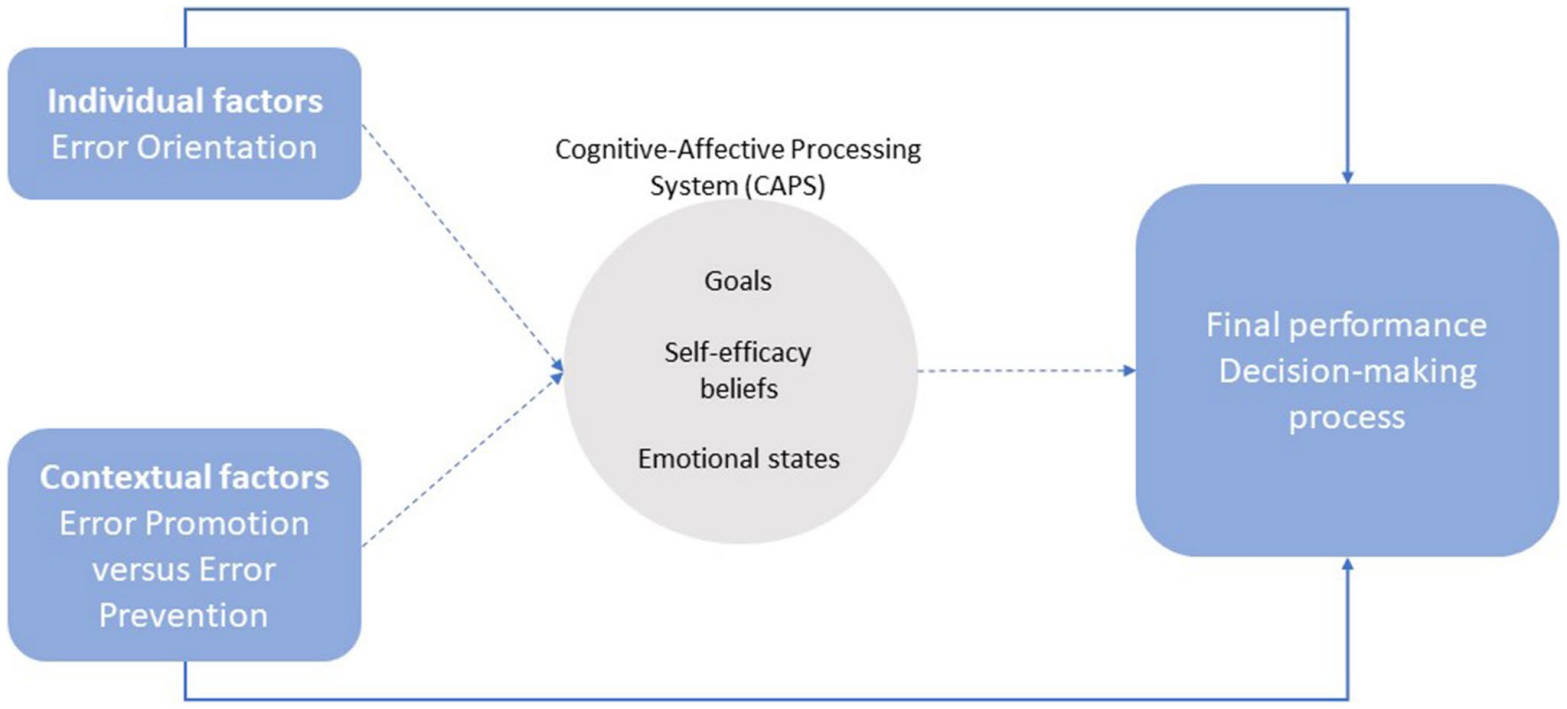
Conceptual framework of the study.

This study is based on the Social Learning Theory as an integrating framework used to analyse how people tackle challenging tasks, how they respond to possible errors, and how personal and situational factors influence their motivation, performance, and learning (Mischel and Shoda, 1995, 1998; Bandura, 1997; Mischel, 2004). From this perspective, situational and dispositional factors influence behavior through cognitive and affective self-regulatory mechanisms, including self-efficacy, which in turn determine the goals that people set for themselves and their affective reactions to the levels of performance achieved (e.g., Tabernero and Wood, 1999). In this model, the impact of the situation on behavior is explained according to how individuals perceive and construct their environment socially (see Figure 1).

### Error orientation and performance

“Errors occur when there is an unintended deviation from a goal or standard” and the factors causing this deviation are potentially avoidable (Frese, 1995; Hofman and Frese, 2011, p. 5). Information about errors is included in many feedback messages individuals receive about their performance providing valid information about how to alter the course of action to achieve that goal. Here, one could also give a definition of error orientation as the way an individual or organization deals with errors determining the amount of learning since, for example, errors used as enhancers of learning lead to better performance (e.g., Dormann and Frese, 1994). In this respect, learning could occur when the individual is motivated to learn from errors, when s/he thinks about these errors metacognitively (planning, assessing, and analysing actions) and when the emotional impact caused by an error is minimized (Heimbeck et al., 2003; Keith and Frese, 2005). Experimental evidence has shown that a positive error orientation associated with the ability to think about the occurrence of errors, the need to communicate them, etc., has a potential positive impact on performance (Frese and Zapf, 1994; Frese, 1995; Nordstrom et al., 1998). Organizations that have an open flow of communication about the constant threats, changes, and strategies to be put in practice regarding possible errors are also stated to be more effective and achieve greater success in the increasingly demanding global market (e.g., Frese and Keith, 2015).

According to studies, error communication is probably the most important error management practice (Van Dyck et al., 2005). Open communication of errors allows for the development of shared knowledge about these errors within the organization (common risk situations, effective coping strategies), as well as fast detection and error management. Furthermore, if the organizational culture focuses on learning from errors, this could also stimulate innovation, given that, as a general rule, innovation involves situations of uncertainty in which errors are highly likely. For innovation to be possible, however, workers should feel free to make such errors and confident that they will not be made to feel guilty or ridiculed as a result (Edmondson, 1999).

Over the last years, some studies have demonstrated the efficacy of error management training, where errors provide informative feedback when they are explicitly incorporated into the training process (Heimbeck et al., 2003; Keith and Frese, 2005; for a meta-analysis, see Keith and Frese, 2008), consequently, training participants are exposed to errors during the training process and are encouraged to use these errors as a learning device by means of positive error statements (Keith and Frese, 2005). Specifically, individuals who focus their goals toward learning and receive this type of training are more willing to take risks (Keith and Frese, 2005; Keith and Frese, 2008; Frese and Keith, 2015). Therefore, certain studies suggest that participants could benefit in different ways from error management or avoidance training, depending on personal characteristics such as cognitive capacity, openness to experience and goal orientation (Hollenbeck et al., 1995a; Gully et al., 2002; Heimbeck et al., 2003).

Some research has analysed the effect of the interaction between personal and contextual factors on self-regulatory mechanisms and analytical strategies that individuals and groups pursue when faced with new and complex tasks, such as organizational decision-making procedures (Tabernero and Wood, 1999; Wood et al., 2000). These authors show that, although the context is decisive initially, in the long term, personal dispositions eventually play a more significant role.

Based on the research analysed, personal dispositions that are linked to motivation to learn from complex tasks, such as error orientation, will be related to performance in the simulation task. We propose the following hypothesis:

*Hypothesis 1:* Error orientation will be significantly and positively correlated with performance in a complex decision-making task.

### Error promotion vs. error prevention: influence of the context

The Self-regulatory Focus Theory proposed by Higgins (1997) essentially refers to the way that people go toward pleasure and shy away from pain. Higgins considers that there are two underlying concepts for the hedonistic pleasure of self-regulation: A *Promotion* self-regulatory focus and a *Prevention* self-regulatory focus (Higgins, 1997, 1998; Idson et al., 2000). The first one involves paying attention to positive results (their presence and absence) and an inclination to go toward the desired end-state as a natural strategy to achieve goals. The *Prevention* focus, on the other hand, implies a special sensitivity to negative results (their absence and presence) and an inclination to avoid the non-achievement of the desired end-state as a natural strategy to achieve goals. Hence, self-regulation with a promotion focus is linked to development, growth, and achievement, motivating individuals to look for gains and avoid non-gains. On the other hand, self-regulation with a prevention focus is linked to protection, security, and responsibility, motivating individuals to ensure the absence of negative results and face the presence of them. According to this theory, situational contexts can also temporarily induce a promotion or prevention focus on the achievement of goals. For example, feedback messages or task instructions can communicate information of gain/non-gain (promotion) or of non-loss/loss (prevention).

Using the same paradigm, Crowe and Higgins (1997) found that when individuals work on a task where generating any number of alternatives is correct, those that have a promotion focus generate a higher number of different alternatives (ensuring successes), whereas those that have a prevention focus are more repetitive (ensuring the avoidance of errors of omission). The results of additional studies provide substantial evidence for the motivation of impulsiveness in the former as opposed to the motivation of vigilance in the latter (Förster et al., 1998; Liberman et al., 1999, 2001). In short, a promotion focus should lead to a more risk-taking style of processing, whereas a prevention focus should lead to a more cautious style of processing, focusing on avoiding errors. Moreover, one fundamental distinction we drew earlier between promotion and prevention motivation is that promotion concerns are rooted in advancement needs, whereas prevention concerns are rooted in security needs. Therefore, those focused on promotion vs. prevention should show a special interest in and sensitivity to information that is particularly relevant for advancement vs. security (Molden et al., 2008).

The self-regulatory focus has been studied as both an orientation that is induced by situations, as something temporary, and as a stable individual factor. In both cases, people who maintain a promotion focus should show a predisposition to give more risky responses, while those who have a prevention focus should show more conservative responses. Since errors and negative feedback about performance are differently considered by people on promotion vs. prevention frames (Idson et al., 2000; Van-Dijk and Kluger, 2004), as opportunities to learn and improve their performance (gains) and as an example of their poor abilities (losses), respectively, we want to explore how people behave in a complex task where errors are encouraged or avoided.

Another important strategic component in the search for goals is the emphasis placed on speed (or quantity) vs. precision (or quality). The regulatory focus theory predicts that, since covering the maximum number of possibilities maximizes the opportunity to achieve success, people with a promotion focus are more likely to stress speed over precision. On the contrary, since scrutinizing the characteristics of a task and the effort exerted minimizes the possibility of errors, people with a prevention focus are more likely to stress precision over speed (Förster et al., 2003). Regarding the research, we propose the following hypothesis:

*Hypothesis 2a:* People who work under an error promotion context will carry out a complex decision-making task faster than those who work in an error prevention context.

*Hypothesis 2b:* People who work under an error promotion context will have better performance than those who work in an error prevention context.

Additionally, the context of errors could also influence the decision-making process. Errors and negative feedback can produce stress and anxiety, partly due to additional demands that individuals who make errors must address. Hence, in organizations in which errors are not punished, but rather accepted as part of the job, additional cognitive demands can be reduced to a limited need for individuals to deal with negative emotional aspects derived from hiding errors or being blamed by others (Hollenbeck et al., 1995b). Furthermore, given that negative information requires more processing (Kanfer and Ackerman, 1989), if the context in which the task is carried out enhances the negative effect of errors on performance, the individual will tend to be self-conscious and concerned about other people’s perception after making the error, which would reduce the attention paid to the actual task itself.

Another implication of the promotion vs. prevention focus involves the strategies used to perform a complex task. Those with a promotion focus may adopt eager decision strategies that emphasize the possibility of gains, whereas those with a prevention focus may adopt more vigilant decision strategies that emphasize the possibility of losses. Finally, the focus on promotion and prevention might also affect the way people cope with the consequences of their decisions: they would need more time to process the negative feedback following their decision and their reasoning (arguments) provided throughout the task would be focused more on their own self-assessment than on improving performance in the future (Goodman et al., 2004; Goodman and Wood, 2004).

*Hypothesis 3a:* People who work in a prevention context will take significantly longer time to make decisions than people in a promotion context.

*Hypothesis 3b:* People who work in a prevention context will show different arguments to explain their decisions (more cautious and self-focused) than people in a promotion context.

Following the theoretical framework proposed, this study analyses the effect of individual and contextual factors on performance and decision-making process in a computer- simulated context of innovation and change.

## Materials and methods

### Participants and procedure

The sample included 40 employees of a Spanish transportation company: 37.5% were women and 62.5% were men. The age range of the sample population was essentially young adult: 40% between the ages of 26 and 35, and 27.5% between the ages of 36 and 45 (these variables were not decisive in the data analysis).

The study was developed in two phases. First, all staff received an internal memo inviting them to participate in the research project. Anyone interested had the opportunity to fill out a questionnaire on the company intranet, which made it easier for employees to access and respond. When the user accessed the questionnaire, the server automatically created a six-digit code, which made it very difficult to identify the participants.

The second phase involved carrying out a complex decision-making task through a multimedia simulator. A simulator created by the INSEAD Business School (Angehrn, 2004)^1^ was used, based on the challenges of change management, technological innovation, and people management in organizations, and which aims to expose the participant to factors that influence the dynamics of innovation and change, elements that are present in all modern organizations. None of the participants had previous experience in the task; therefore, the level of experience of the participants cannot be considered a decisive element in their performance.

Before the employees began the simulation, a DEMO explained each of the tools that they could use during the tasks and the goal that they were trying to achieve: to get as many adopters as possible, a task that they should carry out over 6 months of simulated work. Participants could monitor their progress on the screen and the time it took them to achieve their goal throughout the entire simulation process.

Once they had entered the task context, the system randomly assigned the condition to each participant, trying to balance experimental conditions: Error Promotion vs. Error Prevention. For this purpose, the theoretical framework developed by Higgins et al. (Förster et al., 2003) was used, called the ‘Promotion vs. *prevention focus in self-regulation theory’.* This model, which is strongly present in literature on motivation, makes the distinction, as mentioned previously, between “promotion,” which emphasizes the search for rewards, and “prevention,” which emphasizes security and the avoidance of punishments (Higgins, 1998). That is, an eager strategy to manage errors or negative feedback they could cope with during the task, promoting a positive attitude toward them (e.g., “… you should not be discouraged; keep thinking that you can always achieve a good result”), or a vigilant strategy in front of uncertain decisions they must make throughout the whole simulation (e.g., “… you must be cautious and try to make good decisions, minimizing errors in the process as far as possible”) According to Molden et al. (2008), we should distinguish promotion and prevention concerns from approach and avoidance motivations. So, our experimental manipulation only examined two of the four possibilities of interaction: In the Error Promotion frame, we highlight advancement (gains) and happiness from errors and negative feedback, whereas we underline threat (losses) and anxiety for people who make errors or bad decisions in the Error Prevention frame.

To make this manipulation as credible as possible, the two conditions were built into the software of the simulation program. The manipulation consisted of a preliminary note presented at the start of the simulation and a series of heuristics that appeared in a certain sequence, created specifically for this study, on the simulation program screen. Table 1 shows the preliminary note and the heuristics for each condition. The aim of these notes was to create an attitude of error prevention or promotion in the simulated organization in which they had to act as change managers, in order to, subsequently, study the effect of both frames on the performance of participants.

**Table 1:** Manipulation created to simulate two different organizational frames toward errors: promotion vs. prevention.

Condition: “Error promotion” *Error promotion*	
Preliminary note	Heuristics
*During training, you should expect to make errors. Errors are a positive and essential part of any learning experience. If you make an error, you should not worry, it’s completely normal. Remember that there is always a way to move on after an error and learn from it. This simulation is not an evaluation; it is an example of organizational dynamics. So you WILL NOT BE EVALUATED based on the decisions you make*	H_1_: Remember that you should not rush; focus on all the information available to you H_2_: Remember that you should not worry about errors H_3_: Remember that you should not feel frustrated if your decision is not the most appropriate one H_4_: Remember that you should not be discouraged; keep thinking that you can always achieve a good result H_5_: Remember that we all learn from our errors H_6_: Remember that you do not have to compare yourself with your colleagues H_7_: Remember that this is not an evaluation; there are no better or worse results
Condition: “Error prevention” *Error prevention*	
Preliminary note	Heuristics
*To help you through this simulation program, you have been provided with detailed instructions about how you should carry out your function as a change agent. You can use these instructions at any time during the task*	H_1_: Remember that any error when making decisions could lead to a bad result H_2_: Remember any “bad” decision could affect the overall performance H_3_: Remember that you must be cautious and try to make “good” decisions, minimizing errors in the process as far as possible H_4_: Remember that it is difficult to turn a negative result around (it’s difficult to get out of a negative situation) H_5_: Remember that you must minimize the number of errors in your decisions H_6_: Remember that “bad” decisions take up your working time H_7_: Remember that your errors could affect the image that others have of your performance

As mentioned previously, throughout the entire process, participants chose the strategy to be followed when managing the innovation and change initiatives that they would use to achieve their goals. At any time, the participant could view a detailed summary of previous decisions taken and progress made. Furthermore, they could describe or clarify their motivations for taking each of the decisions throughout the simulation (give arguments). As discussed below, the different elements of the simulation report were divided into quantitative and qualitative data for the analyses.

### Measures

#### Questionnaire

##### Individual error orientation scale

In this study an adapted version of the Error Orientation Questionnaire -EOQ- (Rybowiak et al., 1999) was used. This questionnaire refers to attitudes toward errors and error management in the workplace. We used 37 items from the original scale divided into six subscales following the results obtained in a preliminary study: Learning from Errors, Error Risk Taking, Error Communication, Thinking about Errors, Error Strain, and Covering up Errors. Participants were asked to indicate to what extent they agreed with each of the items using a 5-point Likert scale (1 = “Not at all true” and 5 = “Completely true”). The reliability indexes are shown in Table 3 (similar to those obtained in other studies, e.g., Keith, 2005; Keith and Frese, 2005).

#### Performance in the simulation program

##### Final performance values

The simulation program provided a series of data that can be considered dependent variables for this study. The first measurement was the number of adopters achieved with the simulation program. This was the goal of all program participants; there were 24 people in management positions in the subsidiary company and a maximum of 22 adopters for perfect performance. The program also evaluated the points earned with the initiatives used. The maximum number of points was 243. The number of points was related to the progress that participants made, based on the time they took to make decisions and the number and efficacy of the initiatives they adopted, aspects that are discussed in further detail in the next section. Another measurement taken into account was the real time they took to make decisions and finally the time they took to make a decision after receiving negative feedback.

##### Analysis of the decision-making process

Given the importance of analysing the strategy followed by each of the participants to deal with change, the decision-making task was organized into blocks of trials, which allowed for a more exhaustive analysis of the strategic profile followed by the participants in each experimental condition. Since we expect to find significant differences between the two experimental conditions in the decision-making process, especially in the first execution block, we selected the first 20 decisions by dividing them into 5 blocks of 4 decisions each (the values of each block were obtained by finding the mean of each block). Thus, for each participant we had 5 measurements repeated over time.

###### Quantitative values

Within the decision-making process, four aspects were taken into account: the number of days participants took per decision (each decision takes up a specific amount of time), which provides information about their decision-making style, in other words, choosing decisions that take longer at the beginning indicates a less conservative and more risk-taking style. The second aspect was the progress achieved in this initial performance stage; and the real time taken for each decision (average for each block).

###### Qualitative values

Since the simulation program automatically saved the narrative written reports from each of the sessions conducted, the qualitative information included in the explanatory arguments given by the participants throughout the simulation was analysed following the recommendations defined by the Detailed Event Narrative Analysis (DENA) Codification Manual developed by Amabile et al. (2003).

Each argument was codified in two dimensions with its respective categories. Main event, in other words, a general description of the complete argument; and the affective tone, that is, the general negative or positive nature of the argument. As we can see in Table 2, for the first dimension, the *Main event* of the argument, seven categories were defined in accordance with the Self-Regulatory Focus Theory set forth by Higgins et al. (Higgins, 1997, 1998; Idson et al., 2000).

**Table 2:** Categories defined to analyse the Main Event of the arguments given by participant throughout the whole simulation.

Categories	Meaning
Task	Aspects related with the context, mission, and the way of dealing with the challenge proposed by the simulation program and the note that the participants read at the start of the activity
Strategy	Aspects that the participants commented on when devising their strategy used to deal with the program and which they completed at the beginning of the task
Prior performance	Comments or aspects related to their previous performance, progress made, and errors or errors in decision making
Initiative taken	Aspects related to the actual decision implemented at that time and which describe the purpose of that initiative or elements included in its definition (information which all participants can access at any time during the simulation)
Future events	Aspects related to their expectations about the results to be achieved with the decisions they make and how they can be affected by the dynamics of the simulation in the remaining time
Group dynamics	Elements related to the organization’s working groups, with the different managers and their behavior toward others
Self	Aspects relating to the participants themselves, their state of mind, interest, and motivation in relation to the simulation

The *affective tone* of the arguments was analysed with four emotional categories: positive (the argument highlights pleasant aspects, or a happy mood related to the task), negative (the sentences underline strain or anxiety related to any aspect of the simulation), neutral (the argument does not have a clear emotional side) and ambivalent (participant explains her/his decision both in a negative and positive way) general tone of the statements given by participants.

Two judges, working separately, categorized the arguments of each participant. In most cases, it was not possible to use Cohen’s *Kappa* index to analyse the agreement between the two judges, since this statistic requires a two-way symmetric table in which the values of the first variable are identical to the values of the second. Instead, the Contingency Coefficient was used. This is an association measurement based on chi-square statistics, and the value is always between 0 and 1 (0 indicates that there is no association between the row and the column, while values close to 1 indicate that the variables are closely related). In this study, the average value of the contingency coefficients was 0.75 (range = 0.54–0.88), which indicates a good association between the categorization performed by both judges (Shoukri, 2015).

## Results

### Relationships between error orientation and performance

Regarding Hypothesis 1, as shown in Table 3, the dimension of Error Risk Taking positively correlates with the time taken to carried out the simulation task, and Error Communication positively correlates with the number of adopters achieved as result of the task. Therefore, to maintain a positive attitude aimed at dealing with errors in a suitable way and communicating them, could lead to a more cautious way to carry out the task and, at the same time, to a more successful performance.

**Table 3:** Correlation coefficients between error orientation and performance in the simulation task.

	Mean	SD	1	2	3	4	5	6	7	8	9
Points			–								
Adopters			0.938^**^	–							
Real time			0.162	0.057	–						
Learning from errors	3.89	0.69	−0.534	−0.404	0.062	(α = 0.79)					
Error risk taking	3.82	0.62	−0.307	−0.331	0.680^*^	0.460^**^	(α = 0.74)				
Error strain	2.57	0.68	0.084	0.050	0.188	−0.236	−0.034	(α = 0.77)			
Covering up errors	2.50	0.82	−0.089	−0.215	0.315	−0.321	−0.136	0.391^*^	(α = 0.89)		
Error communication	4.17	0.59	0.445	0.601^*^	−0.097	0.002	−0.038	−0.137	−0.561^**^	(α = 0.71)	
Thinking about errors	4.38	0.67	0.383	0.508	−0.279	0.006	0.114	−0.297	−0.317	0.543^**^	(α = 0.93)

*p < 0.05; **p < 0.01.

### Differences between the experimental conditions in the final simulation performance

**Figure 2:**
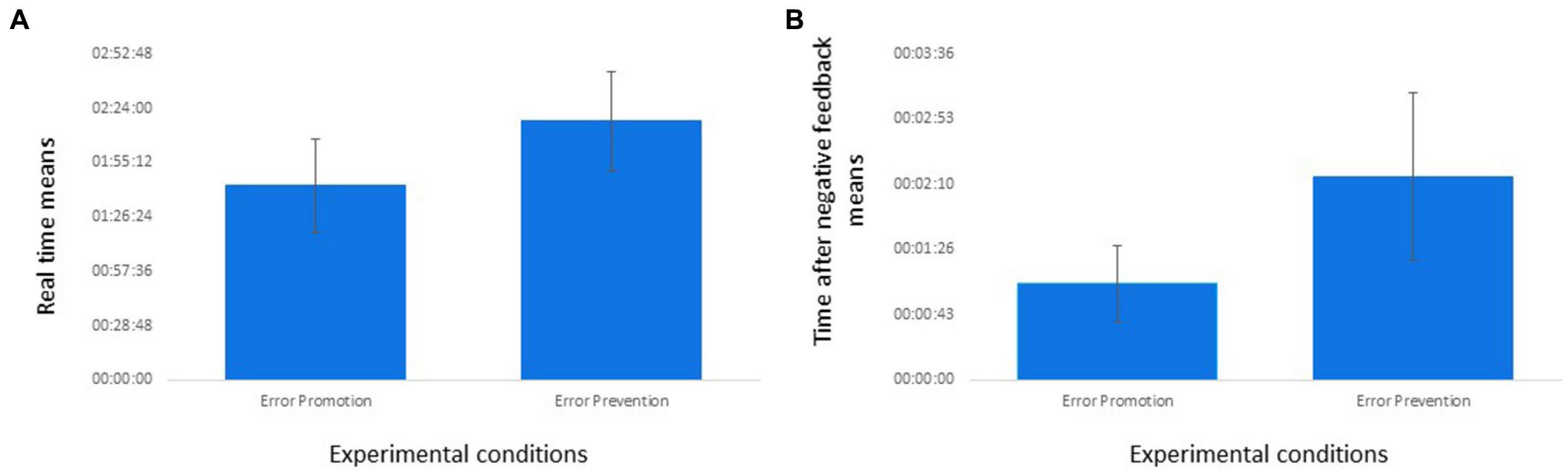
Significant differences between experimental conditions in performance. The part (A) refers to the variable ‘real time’; the part (B) refers to the variable ‘time after negative feedback’.

In relation to Hypothesis 2a and 2b, *t*-tests were carried out. The effect of the experimental manipulation was only significant in the case of the real time the participants took to carry out the simulation [*t*(19) = 2.89, *p* = 0.009]. Participants in the error prevention context took significantly longer to carry out the entire task (see Figure 2A) than participants in the error promotion context. According to the literature, prioritizing speed is a ‘riskier’ strategy focused on maximizing potential gains over time. Therefore, people are more likely to utilize this strategy when pursuing promotion concerns, as our data show. In contrast, prioritizing accuracy is a more ‘cautious’ strategy focused on minimizing potential losses over time. People are more likely to use this strategy when looking for prevention concerns. Although the differences between conditions were not significant in the number of adopters [*t*(19) = −0.50, n.s.] and points [*t*(19) = −0.07, n.s.] achieved at the end of the task, participants in the error promotion frame get more adopters (*M* = 5.50) and points (*M* = 104.5) in the simulation than those in the error prevention frame (*M* = 4.43; *M* = 102.71, respectively). Analysing the time that participants took to make a decision after receiving negative feedback, the results indicated that those working in the prevention condition took significantly longer [*t*(15) = 3.66, *p* = 0.002] than the participants working under the promotion condition (see Figure 2B). These results might indicate a differential decision-making process between participants under the two experimental conditions.^2^

### Effect of the experimental condition on the decision-making process

#### Quantitative values

Since we expected to find significant differences between the two experimental conditions in the decision-making process (Hypotheses 3a and 3b), the first 20 decisions were selected and divided into 5 blocks of 4 decisions. Block 1 contained decisions 1–4; block 2 contained decisions 5–8; block 3 contained decisions 9–12; block 4 contained decisions 13–16; and block 5 contained decisions 17–20; the values of each block were obtained by finding the average for the block.

**Figure 3:**
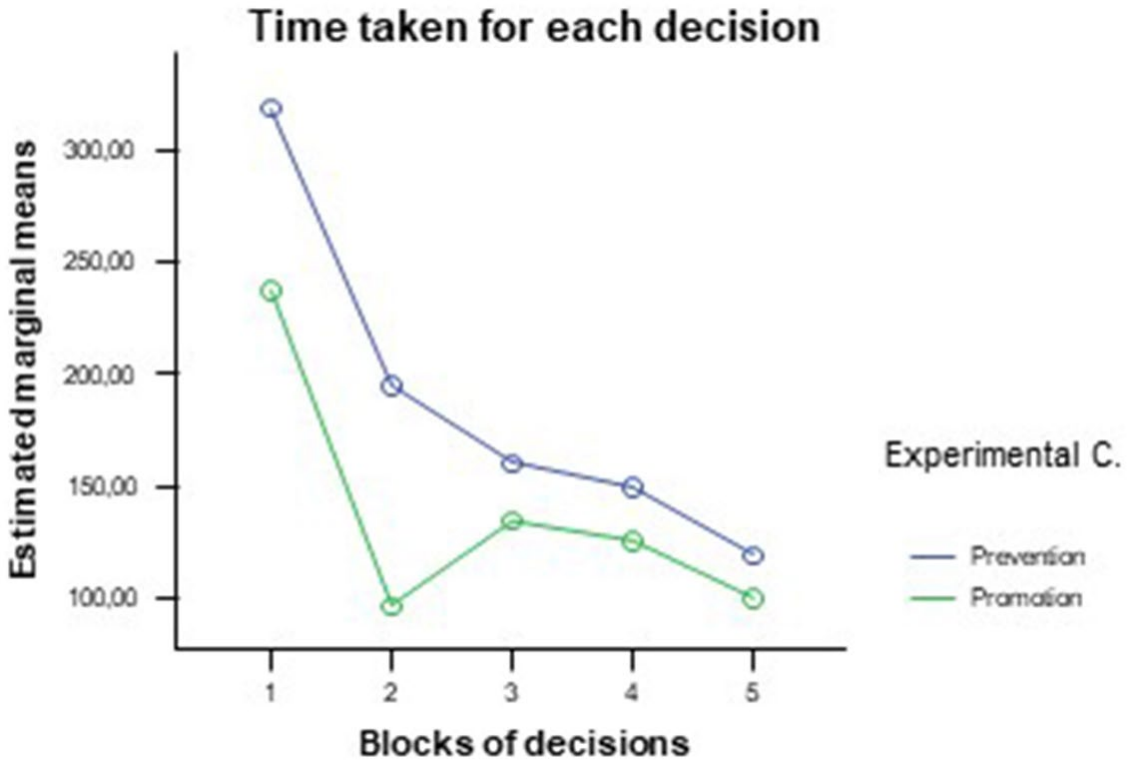
Differences between experimental conditions in the amount of time taken per decision.

The general linear model of repeated measures for the real time measured for each decision taken by the simulation program showed significant differences in the effects of the experimental condition [inter-subject effects, *F*(1, 18) = 5.24, *p* = 0.034; η^2^ = 0.23], whereas the intra-subject effects were not significant: Participants in the error prevention condition took significantly more time to make decisions at this initial stage, showing in this case a “cautious” processing style, according to our hypothesis; however, this tendency appears to fade as the simulation progresses and the two conditions are even out (Figure 3). This could point to a learning effect that would appear for all participants after practicing with the simulation program and could indicate the limited effect of experimental manipulation on initial performance.^3^

#### Qualitative values

First, the number of arguments provided by the participants throughout the program was counted. Although the differences between the two experimental conditions were not significant [*t*(19) = 1.60, n.s.], participants working under the prevention condition (*M* = 27.57) provided more arguments (which would be in line with our hypotheses: a prevention context would favor the tendency to justify one’s actions to a greater degree, especially wrong actions, which would mean that significantly more time would be spent to complete the simulation) than those who carried out the simulation under a promotion condition (*M* = 15.79). This fact is relevant, since there was no difference between the number of decisions that participants in both conditions took throughout the course of the simulation [*t*(19) = 0.034, n.s.]; in other words, participants under the prevention condition tended to choose to clarify their actions to a greater extent.

The number of words used for each argument was also recorded to determine whether there were differences in the elaboration of those arguments. In this case, the differences were again not significant [*t*(19) =0.54, n.s.]; however, once again, participants in the prevention condition tended to expand their arguments to a greater extent (*M* = 203.71) than those in the promotion condition (*M* = 158.79). As mentioned previously, each argument was categorized in relation to the general content to which it referred and the affective tone that the argument might represent in general terms.

### Differences between the two experimental conditions in the *Main Event* and *Affective Tone*

**Figure 4:**
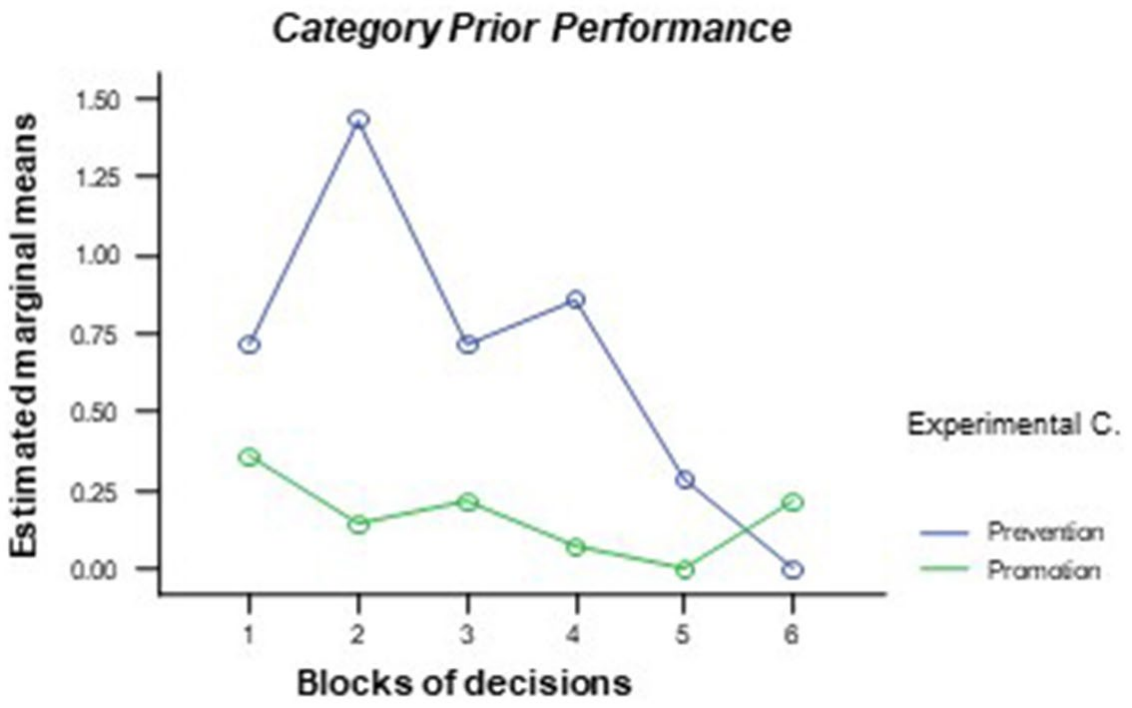
Differences between experimental conditions in terms of the number of times in which the main event of the argument is categorized as *prior performance* through the different decision blocks.

**Figure 5:**
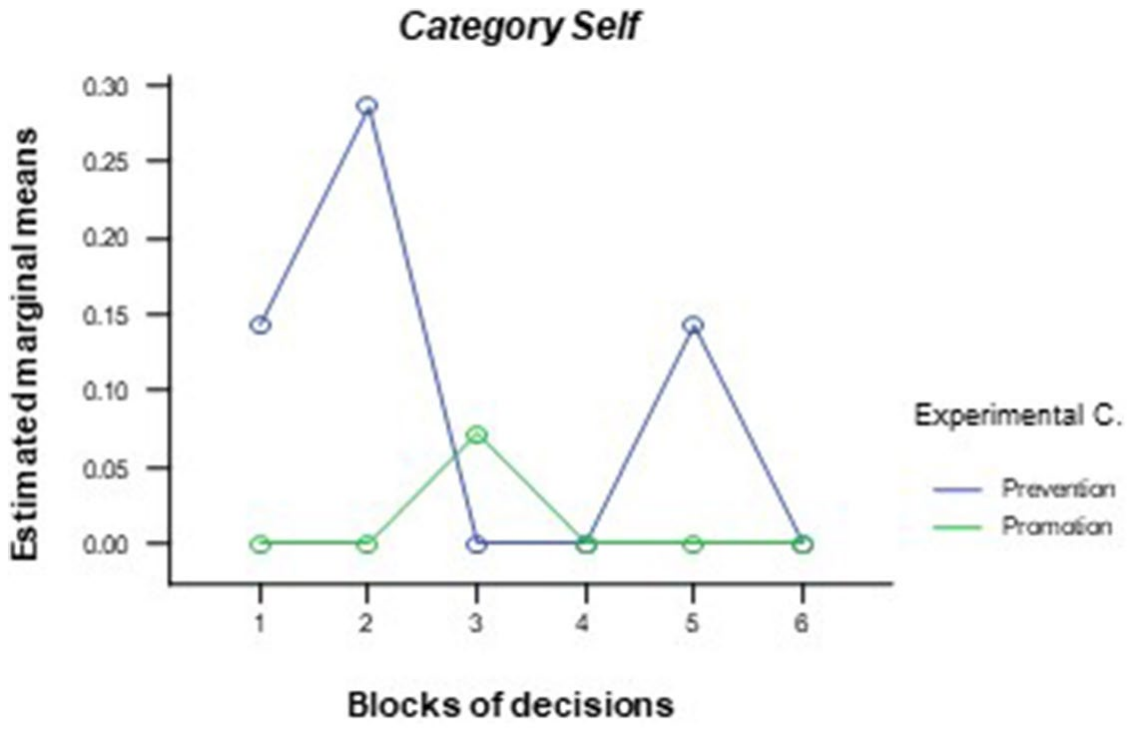
Differences between experimental conditions in terms of the number of times in which the main event of the argument is categorized as *self* through the different decision blocks.

As mentioned previously, the global content of each argument was classified into one of seven categories that refer to the semantic core of that argument (*task, strategy, prior performance, initiative taken, future events, group dynamics*, and *self*). First, *t*-tests were performed for independent samples to explore whether there were significant differences between the two conditions in terms of the total number of appearances for each of the categories. The data confirmed the existence of significant differences in the category *prior performance* [*t*(19) = 2.25, *p* = 0.037] and *self* [*t*(19) = 2.18, *p* = 0.042], which contained more arguments from the participants of the error prevention condition throughout the simulation (*M* = 4.00, sd. = 4.89 and *M* = 0.57, sd. = 0.79, respectively) as opposed to the participants of the promotion condition participants (*M* = 1.00, sd. = 1.04 and *M* = 0.07, sd. = 0.27, respectively). The general linear model of repeated measures for these arguments showed significant differences in the effects of the experimental condition [inter-subject effects, *F*(1, 19) = 5.05, *p* = 0.046; η^2^ = 0.21 for *prior performance* and *F*(1, 19) = 4.77, *p* = 0.048; η^2^ = 0.20 for *self*] (see Figures 4, 5). Thus, participants in the error prevention condition wrote more thoughts about prior performance and self throughout the first blocks of decisions.

The arguments described by the participants were also classified according to the affective tone, into *positive, negative*, *neutral*, or *ambivalent*. No differences were found in the total number of arguments classified in each of the categories.

## Discussion

The aim of this study was to analyse the impact of individual attitudes toward errors and the self-regulatory context on the performance of a decision-making task. The results respond to the need to tackle how people cope with the constant changes that take place in organizations and the role played by certain individual dispositions in the determination of performance in these situations. All of this is linked to the increase in global competitiveness and the fact that organizations and employees nowadays have to be able to adapt to their surroundings quickly.

Results showed that error orientation is related to final performance on a decision-making task, especially error risk taking and error communication. Moreover, regarding the effect of an error prevention vs. an error promotion context, two different experimental conditions were created that allowed the effect on performance in the simulation to be analysed. The effects of the two experimental conditions created were more striking when analysing the time it took participants to make decisions after receiving negative feedback and to complete the simulation program. Those who worked under the *error prevention* condition took significantly longer to perform the task than those who worked under the *promotion* condition. This result is along the same lines as the research conducted by Maule et al. (2000), who considered that in situations where there is time pressure, individuals tend to give greater priority to the processing of negative information. The core issue lies in how the participants constructed that time pressure in relation to the experimental condition. The Social Learning Theory (Mischel and Shoda, 1995, 1998; Mischel, 2004) explains that time pressure can be classed as a threat or a challenge depending on the context (evaluation or learning, respectively). Those working in a prevention condition need more time to process and react, searching for an analysis strategy in the face of negative information. The results support this hypothesis, since when analysing the amount of time employees took to make decisions following a poor performance or having received negative feedback, those working under the prevention condition took significantly longer than those working under the promotion condition.

Since the task was carried out over six simulated months, in which workers had to make decisions to generate change in the organization, this process could be analysed over time. The most significant finding in relation to the differences between the two experimental conditions was observed in the decision-making process developed by each group; in other words, the data about final performance do not seem to be as revealing as the path followed in order to achieve these results.

The results achieved in this study are consistent with those found by Higgins et al. (e.g., Higgins et al., 1997; Idson et al., 2000; Idson and Higgins, 2000), who stress the importance of the motivational context in which a task is performed and its influence on performance. It is possible to situationally induce an error promotion frame vs. an error prevention frame. However, against our expectations, no greater support was found for the effect of experimental manipulation on the final performance of the participants in the simulation program. There are two possible reasons for the absence of such a link: firstly, the positive error orientation of most of the participants might be limiting the effect of the prevention context induced in the initial stages of performance only (participants working under the Error Prevention condition took significantly longer to make decisions, especially in the initial stage, indicating a possible interaction effect between so high dispositional variables and the contextual manipulation created; however, this tendency seems to fade over the course of the simulation, and the two conditions equal out); secondly, the task was designed more as a teaching resource in organizational training programs, rather than a research tool. Hence, the data provided by the program about the performance of the workers are not discriminative enough, and more detailed analysis and control of the variables would be required.

The effect of the error promotion vs. prevention context on performance in simulation is also consistent with the research conducted by Fredrickson (2001), for whom the temporary experience of positive affective states would broaden the thought-action repertoires of individuals (approach, exploration, learning, creativity), while negative affect states would reduce them, leading to defensive behavior (avoidance, escape, attack). This type of approach avoidance behavior is common in our daily lives and in organizations. For example, people can deal with certain tasks by focusing on learning, looking for the intrinsic value in the tasks, and exploring new ways of performing them. At other times, however, they might deal with the same tasks in a defensive way, avoiding punishment. Some authors highlight the importance of affective experience and its role in the self-regulation of the processes involved in making complex decisions in organizational contexts (e.g., Brief and Weiss, 2002).

Prior research suggests that individuals can learn from errors (Keith and Frese, 2005, 2008; Frese and Keith, 2015) and that swift error detection and recovery, as well as open communication and thinking about errors, can have positive implications for organizations (Edmondson, 1996, 1999). The results obtained in this study can shed light on these processes by demonstrating that a situationally induced error promotion vs. prevention frame has an impact on performance and decision-making processes in a complex simulated task. The qualitative data obtained suggest that in a prevention frame, participants provide more information about their motivations for implementing decisions, focusing to a greater extent on their prior performance in the simulation, on aspects relating to their own performance and feelings throughout the task, however, as opposite to our hypothesis, nonsignificant differences were obtained in the affective tone of those statements. In this sense, as Zhao and Olivera (2006) highlight, situational factors, such as time pressure, present in our simulation task as well, are likely to affect error reporting. “As time pressure increases, people use information-processing strategies that demand less cognitive resources” (p. 1027), such as the participants in error prevention condition justifying the decisions made. Following Dahlin et al. (2018) approach, it is necessary to have these three mechanisms to benefit from errors in an organizational setting: the ability, with a positive attitude or error orientation; the motivation, working in a context which promotes learning from errors; and the opportunity to communicate them, also improved by a context with positive frames toward errors.

### Limitations and future research

It is important to review some of the difficulties encountered and conditions that may have impeded to some extent the development of this study, as well as to discuss possible relevant aspects for future research. First, the climate of uncertainty or job insecurity - owing to the time at which the study was performed - must be highlighted; it might have been a stressor or negative factor for the degree of participation of the employees. This study was carried out in an uncertain moment for the company because they were involved in an important merger with other two companies. As literature shows (e.g., Seo and Hill, 2005), this kind of organizational change has important psychological and behavioral effects on employees. One of the weakest points of this study is the number of participants; although the sample was significant in relation to the organization population, a greater number of participants would have been preferable to confirm the hypotheses. Therefore, it would be interesting to perform studies along the same lines, but with a larger sample of employees, from different business units and sections, and employees from different positions, as well as to carry out cross-organizational research in order to expand or clarify the results.

In the new era, organizations face a highly competitive social and economic environment that is constantly changing, as was the case with this organization, which was immersed in a process of job restructuring. Therefore, it would be interesting to see whether future research would confirm the same relationships or to what extent they would change. Furthermore, the effect of other key elements in the organization could be analysed, such as the business unit to which each employee belongs, the position and tasks performed, work climate and satisfaction, sociodemographic aspects, or the professional career of each employee. Similarly, it would be interesting to analyse the communication styles of leaders and the components of the group within the processes through which groups could develop dispositions and self-regulation mechanisms that would promote more effective performance (Zhao et al., 2018; Wilhelm et al., 2019; Klamar et al., 2022).

Further research should analyse which specific self-regulatory processes are particularly important in these types of tasks, given the low level of structuring and the lack of external guidance so often found in organizational contexts. An error promotion context could well help the participants develop emotional control skills (learn to manage the negative feelings caused by the occurrence of errors or after negative feedback) since error promotion frame put them in a positive context, encouraging participants to adopt a positive view of errors. In an error prevention context, on the other hand, they are warned about the negative effects of errors and, therefore, are not prepared to deal with the negative emotional reactions to possible failures. Furthermore, following on from Keith and Frese (2005), the cognitive control or metacognition processes involved in planning, monitoring, and assessing one’s own progress should be analysed, since they can also be encouraged in an error promotion context.

Another important issue to address could be how time constraints in the simulation program affect strategies followed by participants and changes in the decision-making process, as some authors suggest that under time constraint people use strategies that are easier or more familiar (Ordóñez and Benson, 1997). In this sense, Betsch et al. (2004) state that choices under time pressure will reflect prior learning rather than new behavioral intentions, since cognitive control depends on the number of cognitive resources (i.e., time pressure, constraints cognitive capacity). Moreover, it is important to consider temporal dynamics of errors (Lei et al., 2016) in order to better understand the processes involved and assess causality.

In future studies, it would be interesting to analyse whether an error promotion vs. prevention context could lead to differences in the development and modification of the strategies developed by participants, both explicitly and implicitly, at the beginning of a complex task. On the one hand, a promotion context could foster a high perception of one’s own ability to deal with errors and negative feedback that could occur when working on new tasks. From a practical perspective, it would be important to identify which mediators are involved in the relationship between the context in which the task is performed and the performance, as a way of determining which aspects would be important in the learning and performance of a complex task. It would be relevant to emphasize the critical role of information processing which is promoted by the context in which the task is performed.

In this study we have presented some ways in which promotion and prevention motivations could have effects on performance and decision-making process. Although our results show non-consistent effects, mainly because of the sample size problem discussed above, considering these effects as a whole, it may be tempting to ask, is one motivational orientation ‘better’ than the other? That is, are there greater benefits and fewer costs associated with a promotion or prevention frame related to errors? When comparing promotion and prevention motivations, it may be more accurate to characterize such motivations as involving a series of complementary compromises. A promotion frame prioritizes flexibility, openness, and rapid and eager progress, but does so by sacrificing commitment, certainty, and careful and vigilant analysis. A prevention focus reverses these priorities and sacrifices. All these qualities are important components of self-regulation and goal pursuit, and all are required for the successful execution of these processes. Thus, the most crucial factor may be whether promotion or prevention best fits the demands of the task at hand (Molden et al., 2008).

## Data availability statement

The raw data supporting the conclusions of this article will be made available by the authors, without undue reservation.

## Ethics statement

Ethical review and approval was not required for the study of human participants in accordance with the local legislation and institutional requirements. Written informed consent from the participants was not required to participate in this study in accordance with the national legislation and the institutional requirements.

## Author contributions

EB: conceptualization, methodology, and project administration. CT: writing, review and editing, conceptualization, methodology, and project administration. AA: writing, review and editing, conceptualization, methodology, and project administration. All authors contributed to the article and approved the submitted version.

## Funding

This research was funded by a project of the Spanish Railway Transport Company (GIF) and a project from Instituto de Prevención de Riesgos Laborales (IAPRL), Junta de Andalucía CTC-2022132186).

## Acknowledgments

We acknowledge Professor Sabine Sonnentag for her helpful comments in previous versions of this manuscript. We are grateful to Professor Albert A. Angehrn (INSEAD) who provided access to the simulation program and help in the programming of the experimental conditions.

## Conflict of interest

The authors declare that the research was conducted in the absence of any commercial or financial relationships that could be construed as a potential conflict of interest.

## Publisher’s note

All claims expressed in this article are solely those of the authors and do not necessarily represent those of their affiliated organizations, or those of the publisher, the editors and the reviewers. Any product that may be evaluated in this article, or claim that may be made by its manufacturer, is not guaranteed or endorsed by the publisher.

## Footnotes

^1^For more information about the simulation, please visit https://mycalt.insead.edu/eis/ and https://www.alphasimulation.com/eis-1

^2^Following a reviewer’s comment, the Bonferroni correction to the significance threshold was applied (i.e., for the effect of experimental condition to be significant, its *p* value would have to be less than 0.05/4, or 0.0125) (Cohen and Cohen, 1983). When the more conservative Bonferroni method is followed, only two effects of the experimental condition were significant.

^3^Following a reviewer suggestion, the clustered robust standard errors were considered in a new analysis (Cameron and Miller, 2015). In this case the results differ from those of the general linear model of repeated measures for the real time measured for each decision taken by the simulation program. Taking robust standard errors, the effect of the experimental condition is non-significant.
